# Effect of career calling on learning engagement among medical students: moderated chain mediation model

**DOI:** 10.3389/fpsyg.2026.1684012

**Published:** 2026-03-11

**Authors:** Xiaoguang Wu, Yafei Liu, Wenbin Wang, Zhaoliang Wang, Siyu Di

**Affiliations:** 1 Clinical College of Anhui Medical University, Hefei, Anhui, China; 2 Health Humanities Research Innovation Team, Clinical College of Anhui Medical University, Hefei, Anhui, China; 3 Xinjiang Education Institute, Ürümqi, Xinjiang, China

**Keywords:** achievement motivation, career calling, learning engagement, medical students, self-efficacy

## Abstract

**Purpose:**

This study aimed to investigate the effects of career calling on the learning engagement of medical students, along with the psychological mechanisms of self-efficacy and achievement motivation in the relationship between them.

**Methods:**

The study used a convenience sampling method to select 1,930 students from two undergraduate medical colleges in Anhui Province, China. The Career Calling Scale, Learning Engagement Scale, Achievement Motivation Scale, and Self-Efficacy Scale were used to conduct the survey.

**Results:**

The results of the study found that career calling significantly and positively predicted the learning engagement of medical students. In addition, achievement motivation and self-efficacy not only partially mediated the relationship between career calling and learning engagement but also played a chain mediation role between them. Finally, there were significant gender differences in the above chain mediation model, and gender moderated the path from achievement motivation to learning engagement in the model.

**Conclusion:**

Self-efficacy and achievement motivation act as chain mediators between career calling and learning engagement of medical students, and this path is moderated by gender. This study provides theoretical guidance and empirical evidence for improving the learning engagement of medical students and promoting the development of medical education.

## 1 Introduction

Learning engagement (LE) is a positive and fulfilling emotional, cognitive, and behavioral state associated with learning activities that is enduring and diffuse and is characterized by vigor, dedication, and focus (Schaufeli et al., 2002). Learning engagement not only affects an individual’s motivation to learn but also determines the level of academic achievement and skills an individual can attain (Kuh, 2009). For medical students, the level of their learning engagement directly determines their theoretical foundation and practical ability, which indirectly affects the life and health security of patients. Therefore, improving the level of learning engagement of medical students is necessary to improve the quality of medical student education and promote the development of medical education.

However, the current level of medical students’ learning engagement still leaves a large room for improvement (Agarwal et al., 2019; Gómez et al., 2015; Xia et al., 2023; Xu et al., 2022). Liu et al. (2018), in their investigation of learning engagement among medical students in China, found that the overall learning engagement level of medical students was medium and still has considerable room for improvement, which is in line with the subsequent findings of Huang et al. (2023) and Shida (2024). In addition, survey reports from Portugal (Abreu Alves et al., 2022), Chile (Gómez et al., 2015), Canada (Babenko et al., 2018), the Netherlands (Wissing et al., 2022), and Spain (Urrizola et al., 2023) have also obtained similar results. Therefore, exploring the formation mechanism of medical students’ learning engagement is of great significance for further improving the level of their learning engagement and enhancing their learning initiative.

### 1.1 Relationship between career calling and learning engagement

Career calling (CC) refers to the strong positive emotional experience an individual has when engaging in a career (Dobrow and Tosti-Kharas, 2011). Individuals with a career calling regard their careers as an integral part of their personal lives and work hard not only to obtain external benefits but also to integrate work into their lives as their life goals (Dik et al., 2009). Individuals realize the value and meaning of life through work and help others directly or indirectly to gain a greater sense of satisfaction, which further enhances their positive evaluation and cognitive experience of the occupation (Dik and Duffy, 2007). Although there are fewer current direct studies on medical students’ career calling and learning engagement, a positive correlation between teacher training students’ career calling and learning engagement was found in an investigative study (Chen et al., 2016). Wang et al. (2023) similarly found a positive relationship between career calling and learning engagement among nursing students when they investigated the relationship between the two.

According to the social–occupational cognitive theory, occupational performance is influenced by both outcome expectancy and efficacy expectancy, which in turn are affected by personal traits and environmental factors (Lent et al., 2000). Therefore, occupational performance may be indirectly influenced by such environmental factors. Career calling embodies these environmental factors and represents occupational attitudes formed within specific economic, social, and cultural contexts (Dik et al., 2009). Individuals with a strong career calling understand the value and significance of professional behavior, demonstrating a greater willingness to invest effort in enhancing job performance and realizing personal fulfillment. In summary, Hypothesis 1 proposes that medical students’ career calling significantly and positively predicts their level of learning engagement.

### 1.2 Mediating role of self-efficacy

Self-efficacy (SE) refers to students’ subjective judgment of their ability to successfully complete tasks (Bandura, 1989). Individuals with career calling experience positive emotions during occupational activities, which in turn enhance their self-efficacy (Hu et al., 2024). Consequently, a significant correlation may exist between career calling and self-efficacy, with existing studies supporting this perspective. Seidman et al. (2024) reported a significant positive correlation between healthcare workers’ work engagement and career calling. Furthermore, students with high self-efficacy are more likely to persevere and exert greater effort when confronting academic challenges. Consequently, a significant correlation should exist between learning engagement and academic self-efficacy (Kaufmann et al., 2022). This relationship is supported by existing studies. Stan et al. (2022) and Luo et al. (2023) identified a positive correlation between college students’ learning engagement and self-efficacy.

Social occupational cognitive theory posits that self-efficacy influences individual occupational performance. Self-efficacy is influenced by environmental factors, including an individual’s career calling (Lent et al., 2000). Although no studies have directly demonstrated the mediating role of self-efficacy between career calling and learning engagement, based on social–occupational cognitive theory and related studies, we propose that career calling may indirectly affect learning engagement through self-efficacy. Accordingly, Hypothesis 2 proposes that self-efficacy mediates the relationship between career calling and learning engagement.

### 1.3 Mediating role of achievement motivation

Achievement motivation (AM) is an individual’s subjective expectation of behavioral success or tendency to pursue success. Individuals with a strong career calling perceive the value inherent in professional conduct, thereby exhibiting greater aspiration to achieve career goals. Consequently, a significant positive correlation should exist between career calling and achievement motivation. In studying the influence of career calling and achievement motivation on college students’ career decisions, Yang et al. (2022) found a positive correlation between career calling and achievement motivation, indicating that stronger achievement motivation among college students also enhances their career calling. Moreover, achievement motivation heightens individuals’ expectations regarding task outcomes, and positive outcome expectations influence ultimate task performance (Kiuru et al., 2020). Consequently, learning engagement—as one outcome of learning performance—is influenced by achievement motivation (Sun et al., 2023). For instance, Li et al. (2024) conducted a study on the relationship between learning engagement and achievement motivation among Chinese adolescents. They found a significant positive correlation between the two, specifically demonstrating that stronger achievement motivation among students leads to increased levels of learning engagement.

According to expectancy-value theory, when an individual’s achievement motivation exceeds their avoidance motivation, they are more willing to engage in actions to achieve success. Achievement motivation is influenced by task value; that is, when a task holds high value, individuals become more eager to succeed (Atkinson, 1957). Career calling provides individuals with a sense of value in engaging in occupational behavior. Although no direct studies have examined the role of achievement motivation in mediating the relationship between career calling and learning engagement, based on expectancy-value theory and existing studies, we propose that career calling influences learning engagement by affecting achievement motivation. Accordingly, Hypothesis 3 proposes that achievement motivation plays a mediating role between career calling and learning engagement.

### 1.4 Chain mediation of self-efficacy and achievement motivation

First, medical students with a sense of career calling can recognize a sense of meaning and identity in their learning process, resulting in a positive emotional experience (Zhao et al., 2024). This positive emotional experience will further enhance students’ self-efficacy (Hu et al., 2024). Second, students with high self-efficacy perceive themselves as capable of accomplishing learning tasks and have positive expectations of learning outcomes (Kristensen et al., 2023), which enhances their learning motivation, that is, increasing achievement motivation. Studies have demonstrated a significant positive correlation between self-efficacy and achievement motivation in nursing, physical education, and military students (Cheng et al., 2022; Dan et al., 2023; Li et al., 2021). Finally, students with high achievement motivation exhibit a strong desire for academic success, and they are willing to put in more effort to achieve academic success (Li et al., 2024), which in turn leads to increased learning engagement.

The expectancy-value theory posits that achievement motivation is influenced not only by the value of the task but also by expectations (Atkinson, 1957), with self-efficacy representing the expectancy of efficacy. Therefore, this study concluded that career calling, as a strong and positive emotional experience, effectively increases students’ self-efficacy and achievement motivation, which in turn increases their level of learning engagement. Accordingly, Hypothesis 4 proposes a chain mediating effect of self-efficacy and achievement motivation between career calling and learning engagement.

### 1.5 The moderating role of gender

The impact of gender differences on students’ learning engagement is an issue that cannot be ignored. Shida (2024), in a study on the level of learning engagement of medical students, found that male students have a significantly higher levels of learning engagement than female students. Meanwhile, gender differences have a significant impact on career calling. DiRenzo et al. (2021) investigated the career calling of military personnel of different genders in the U.S. Army and found that men have a stronger career calling than women. In addition, self-efficacy was similarly affected by gender. Luo et al. (2023) investigated self-efficacy among college students and found that male students had significantly higher self-efficacy than female students. Finally, achievement motivation is no exception, with significant differences in achievement motivation among students of different genders (Fischer et al., 2012). In summary, Hypothesis 5 proposes that the chain mediation mechanism through which career calling influences the learning engagement of medical students through self-efficacy and achievement motivation may be moderated by gender.

### 1.6 Theory foundation

Social Cognitive Career Theory (SCCT) emphasizes the influence of self-efficacy and outcome expectancy on individual career goal choice and performance, which are influenced by learning experiences (Lent et al., 2000). Factors affecting individual learning experiences are categorized into two types: personal input and environmental influence. Among them, career calling, as an acquired career attitude influenced by multiple factors, such as economic, social, cultural, and family factors, is determined by the environmental context in which an individual lives (Dik et al., 2009). This finding suggests that career calling can influence individual career performance by affecting self-efficacy. In the case of medical students, it is the sense of career calling that affects their learning engagement by influencing their self-efficacy.

Second, we must not forget the impact of motivation on learning performance. Expectancy-value theory suggests that motivation is divided into two categories: achievement motivation, which maximizes satisfaction for the individual, and avoidance motivation, which minimizes the experience of pain for the individual. Individuals with stronger achievement motivation than those with avoidance motivation are more conducive to successful goals (Atkinson, 1957). For students, individuals with high achievement motivation are more willing to invest time and effort in learning engagement to achieve academic success. Individual achievement motivation is influenced by expectations and values. Expectations are subjective judgments about the likelihood of succeeding in a task and are influenced by self-efficacy. Individuals with high self-efficacy perceive themselves as capable of accomplishing tasks and therefore have higher achievement motivation (Wigfield and Eccles, 2000). Values are stable, holistic beliefs regarding what is worth having, and these beliefs are influenced by social standards, psychological needs, and self-perception. Individuals with a high career calling are better able to understand the value attached to their professional behavior and are clear about what they are supposed to be doing; as a result, their achievement motivation is higher (Eccles and Wigfield, 2002).

Although existing research acknowledges the positive impact of career calling on learning engagement, further investigation is needed regarding the specific pathways linking career calling to learning engagement. This gap affects the practical effectiveness of enhancing medical students’ learning engagement by fostering career calling during medical education. Therefore, clarifying the potential pathways between career calling and learning engagement holds significant practical importance. According to Social Cognitive Career Theory and expectancy-value theory, self-efficacy and achievement motivation may mediate the relationship between career calling and medical students’ learning engagement. Furthermore, due to sociocultural influences, significant gender differences exist in career calling, learning engagement, self-efficacy, and achievement motivation. Thus, when examining the role of self-efficacy and achievement motivation in mediating the relationship between career calling and learning engagement, gender should be considered and analyzed separately.

This study aimed to investigate the influence of career calling on learning engagement among medical students and to understand the roles of self-efficacy, achievement motivation, and gender in the relationship between career calling and learning engagement. This study adopted a questionnaire method using the Career Calling Scale, Achievement Motivation Scale, Learning Engagement Scale, and Self-Efficacy Scale to investigate 1,930 medical students in Anhui Province, China, and to test the following five hypotheses:

Hypothesis 1: Medical students’ career calling can significantly and positively predict their level of learning engagement.

Hypothesis 2: Self-efficacy mediates the relationship between career calling and learning engagement.

Hypothesis 3: Achievement motivation mediates the relationship between career calling and learning engagement.

Hypothesis 4: There is a chain mediating the role of self-efficacy and achievement motivation between career calling and learning engagement.

Hypothesis 5: The chain mediating mechanism of career calling influencing learning engagement of medical students through self-efficacy and achievement motivation may be moderated by gender.

## 2 Materials and methods

### 2.1 Participants

Using cluster sampling, electronic questionnaires were distributed to medical students at two undergraduate medical universities in Anhui Province, China, randomly selected by grade level. The combined enrollment of both institutions in 2024 was approximately 28,000 students. To assess cluster effects, intraclass correlation coefficients were tested. The results indicated that the ICC for career calling was −0.01, achievement motivation was 0.11, learning commitment was 0.11, and self-efficacy was 0.11—all falling within the range of low or no consistency (Shrout, 1998). This suggests a weak cluster effect that does not significantly impact the precision of standard error estimates, eliminating the need for multilevel linear model correction.

To protect participant privacy, the electronic questionnaire does not contain any content related to personal information. To prevent missing data, all items are set as mandatory questions. Only after completing all items can the electronic questionnaire be submitted. Data screening criteria are as follows: (1) based on existing research experience, the response time for each question should not be less than 2 s (Huang et al., 2012). With 58 questions in total, the total response time should be no less than 116 s. Strictly speaking, questionnaires with response times under 120 s are classified as rushed responses and discarded. Such questionnaires often indicate careless responses or failure to read questions carefully. (2) patterned response questionnaires were also excluded; these were defined as those where respondents consistently select the same option or follow a fixed, repetitive sequence. Such response patterns indicate that participants did not respond based on their genuine circumstances, rendering the data invalid.

A total of 2,172 questionnaires were collected, with 1,930 valid responses yielding an 88.9% response rate. The sample size represents approximately 6.9% of the population. The non-response rate was 11.1%, comprising 61 participants who completed the questionnaire at a regular pace and 181 who completed it quickly. To assess baseline consistency between the included and excluded samples, demographic variables, such as gender, grade level, and mean age, were compared between the two groups. The results revealed that the included group comprised 756 men (39.2%) and 1,174 women (60.8%). The exclusion group comprised 97 men (40.1%) and 145 women (59.9%). The gender distribution difference between groups was not statistically significant (*χ^2^* = 0.08, *p* = 0.78). The inclusion group comprised 608 first-year students (31.5%), 1,034 s-year students (53.6%), and 288 third-year students (14.9%). The exclusion group included 66 first-year students (27.3%), 128 s-year students (52.9%), and 48 third-year students (19.8%). The distribution of grades between the two groups showed no statistically significant difference (*χ^2^* = 4.61, *p* = 0.10). The mean age in the inclusion group was 20.11 (SD = 1.66), while that in the exclusion group was 20.24 (SD = 1.85). The difference in age between the two groups was not statistically significant (*t* = 1.10, *p* = 0.27). The results indicate that baseline characteristics were balanced and comparable between the inclusion and exclusion groups, suggesting a minimal impact of response bias on study outcomes.

The sample consisted of 756 men and 1,174 women, including 608 first-year students, 1,034 s-year students, and 288 third-year students, with an average age of 20.11 years (SD = 1.66). This study was conducted in accordance with the Declaration of Helsinki.

### 2.2 Materials

#### 2.2.1 Career calling

The Career Calling Scale, developed by Zhang (2015) in Chinese culture, was adopted. The scale comprises 11 questions. Participants answered each item on a five-point Likert scale (1 = totally disagree, 5 = totally agree). Higher total scores (reverse questions were transformed) indicate a higher career calling. The scale comprises three dimensions: altruism, guiding force, and meaning and purpose. Altruism emphasizes the desire to provide assistance, serve others or the society, and the spirit of dedication. The guiding force highlights career calling as a compelling force or significant undertaking that compels individuals to embrace and strive to fulfill it. The meaning and purpose dimension reflects the integration of one’s professional role with personal life, meaning, purpose, and interests. In this study, Cronbach’s alpha was used. The Cronbach’s alpha coefficients for the three dimensions were 0.74 (altruism), 0.89 (guiding force), and 0.89 (meaning and purpose). Structural validity was favorable: *χ^2^/df* = 6.35, TLI = 0.98, CFI = 0.99, RMSEA = 0.05, and SRMR = 0.03.

#### 2.2.2 Learning engagement

The Learning Engagement Scale for College Students, developed by Liao (2011), was used to investigate medical students’ learning engagement. The scale comprises three dimensions: behavioral, cognitive, and emotional engagement. Behavioral engagement (BE) refers to students’ classroom performance, extracurricular learning engagement, and professional practice. Cognitive engagement (CE) refers to students’ adoption of learning methods and strategies based on their knowledge of the profession. Emotional engagement (EE) refers to the emotional experience of college students when learning professional knowledge. The scale consists of 20 items. Participants answered each item on a five-point Likert scale (1 = totally out of line, 5 = fully in line). Higher scores indicate higher learning engagement. In this study, the Cronbach’s alpha of the scale was 0.95. The Cronbach’s *α* of the subscales was 0.86 for BE, 0.93 for CE, and 0.88 for EE.

#### 2.2.3 Self-efficacy

The Self-Efficacy Scale, adapted from Zhang and Schwarzer (1995), was used. The scale comprises 10 items. Participants answered each item on a four-point Likert scale (1 = completely disagree, 4 = completely agree). Higher total scores indicate higher self-efficacy. The Cronbach’s *α* for this scale in this study was 0.77.

#### 2.2.4 Achievement motivation

The Achievement Motivation Scale, revised by Ye and Hagtvet (1992), was used in this study. The scale comprises 15 items. Participants answered each item on a four-point Likert scale (1 = totally out of line; 4 = fully in line). Higher total scores indicate greater motivation for student achievement. The Cronbach’s alpha for the scale in this study was 0.92.

### 2.3 Statistical processing and analysis plan

First, data analysis was conducted using SPSS 21.0. Valid questionnaires were retained, defined as those with consistent item selections and completion times under 120 s. Missing data were imputed using maximum likelihood estimation. Second, scores for career calling, learning commitment, achievement motivation, and self-efficacy were standardized. Finally, data analysis was conducted using Amos 24.0 with the PROCESS plugin. PROCESS 4.1 was employed to examine chained mediation effects, with results presented in Section 3.3. Amos 24.0 was used to test the moderating effect of gender on chained mediation, with findings detailed in Section 3.4.

Structural equation models were constructed by grouping the participants by gender. Each group comprised ten observed variables. Career calling was divided into three observed variables: altruistic contribution, orientation, and meaning and value. Learning engagement was divided into three observed variables: behavioral, cognitive, and emotional engagement. Self-efficacy and achievement motivation were each grouped into two variables based on their factor loadings. The total number of estimated parameters was 59. Maxwell (2000) suggests that the sample size should be 20 times the number of estimated parameters in the model. Thus, the required sample size for this study was 1,180, indicating that the current sample size met the necessary condition.

## 3 Results

### 3.1 Common method bias control and test

An unrotated exploratory factor analysis of the 56 items was conducted using Harman’s one-way test. The results showed that there were nine factors with eigenvalues greater than 1 when unrotated, explaining 64.37% of the total variance. The first factor explained 33.95% of the variance, which was less than 40%. Therefore, the results of this study were less affected by common method bias.

### 3.2 Descriptive statistics and correlation analysis

The descriptive statistics of each variable are shown in Table 1. The results of the correlation analysis showed that there was a significant positive correlation between career calling, self-efficacy, achievement motivation, and learning engagement.

**Table 1:** Descriptive statistics and correlation analysis (*n* = 1,930).

	M	SD	1	2	3	4	5	6	7	8
Grade	1.83	0.66	—							
Gender	1.61	0.49	−0.12^**^	—						
CC	41.43	5.99	−0.12^**^	−0.06^**^	−0.07^**^	−0.08^**^	—			
AM	40.95	7.62	−0.08^**^	−0.15^**^	−0.02	−0.03	0.59^**^	—		
LE	64.68	13.45	−0.05^**^	−0.08^**^	−0.06^*^	−0.04	0.48^**^	0.56^**^	—	
SE	47.30	6.53	0.07^**^	−0.00	−0.02	−0.05^*^	0.37^**^	0.45^**^	0.61^**^	—

^*^p < 0.05, ^**^p < 0.01. Male = 1, Female = 2; CC = career calling; AM = achievement motive; LE = learning engagement; SE = self-efficacy.

### 3.3 Analysis of the chain mediation effect

**Figure 1:**
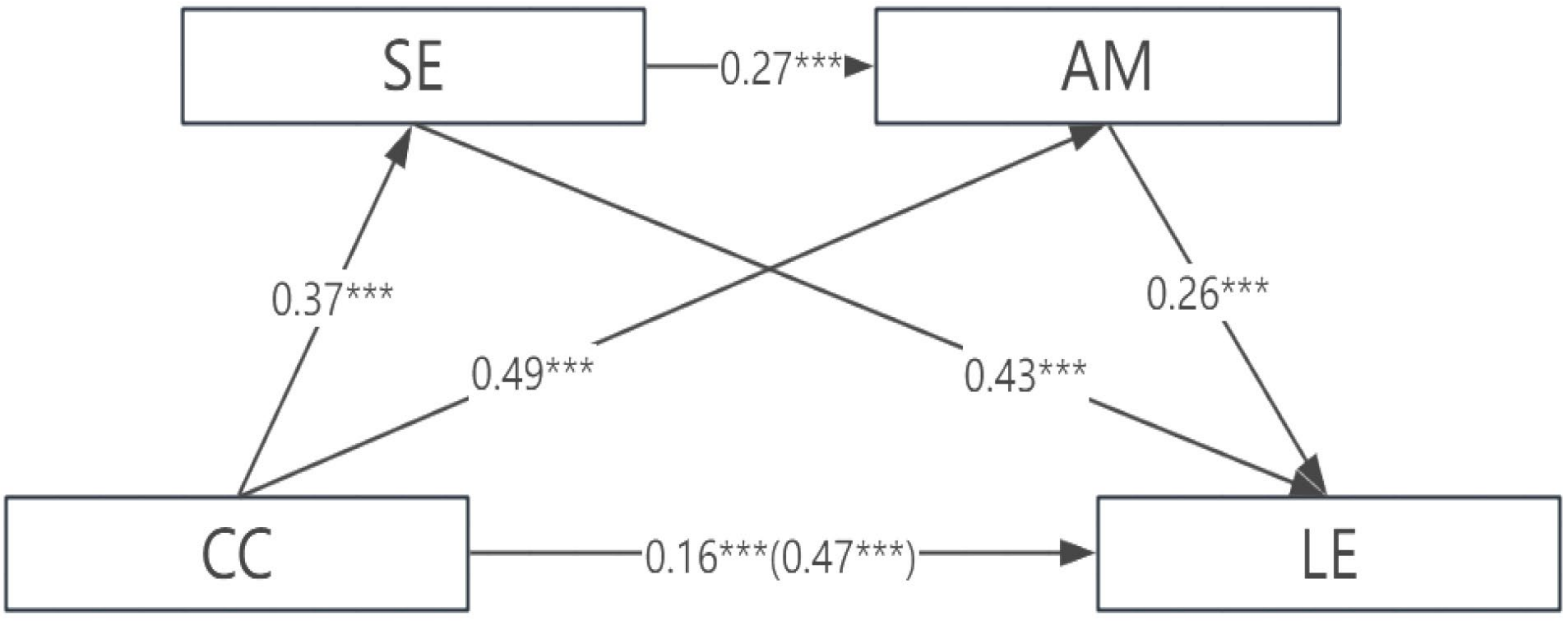
Path diagram for testing the chain mediation effect.

Regression analysis results indicate that career calling significantly and positively predicts learning engagement (*β* = 0.47, *p* < 0.001). After incorporating two mediating variables, career calling significantly and positively predicted achievement motivation (*β* = 0.49, *p* < 0.001) and self-efficacy (*β* = 0.37, *p* < 0.001). Self-efficacy significantly and positively predicted achievement motivation (*β* = 0.26, *p* < 0.001) and learning engagement (*β* = 0.49, *p* < 0.001). Achievement motivation significantly and positively predicted learning engagement (*β* = 0.27, *p* < 0.001), while career calling continued to significantly and positively predict learning engagement (*β* = 0.16, *p* < 0.001). See Table 2, Figure 1.

**Table 2:** Model analysis of chain mediation.

Result variables	Predictive variables	*R*	*R^2^*	*F*	*β*	*t*	95% CI
SE	CC	0.37	0.14	100.75	0.37	17.08^***^	[0.32, 0.41]
	Grade				−0.03	−1.03	[−0.10, 0.30]
	Gender				0.03	0.74	[−0.06, 0.12]
AM	CC	0.66	0.43	361.72	0.49	25.98^***^	[0.45, 0.52]
	SE				0.27	14.48^***^	[0.23, 0.31]
	Grade				−0.04	−1.37	[−0.09, 0.02]
	Gender				−0.26	−7.26^***^	[−0.33, −0.19]
LE	CC	0.70	0.49	364.49	0.16	7.90^***^	[0.12, 0.20]
	SE				0. 43	23.24^***^	[0.39, 0.47]
	AM				0. 27	12.21^***^	[0.22, 0.31]
	Grade				0.03	1.26	[−0.02, 0.08]
	Gender				−0.06	−1.69	[−0.13, 0.01]

AM, achievement motive; CC, career calling; SE, self-efficacy; LE, learning engagement. ***p < 0.001.

### 3.4 Analysis of moderating effects

Using AMOS 24.0, with grade level as a control variable and gender as a grouping variable, the differences in the mediating effects of self-efficacy and achievement motivation between career calling and learning engagement across genders were analyzed.

**Figure 2:**
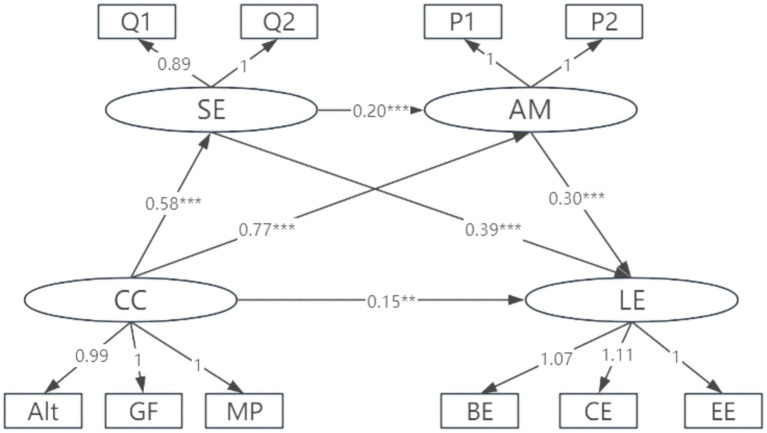
Chain mediation effects of achievement motivation and self-efficacy (male students). BE, behavioral engagement; CE, cognitive engagement; EE, emotional engagement; P1–P2, S1–S2, Q1–Q2 represent the packaged dimensions of achievement motivation, career calling, and self-efficacy, respectively.

**Figure 3:**
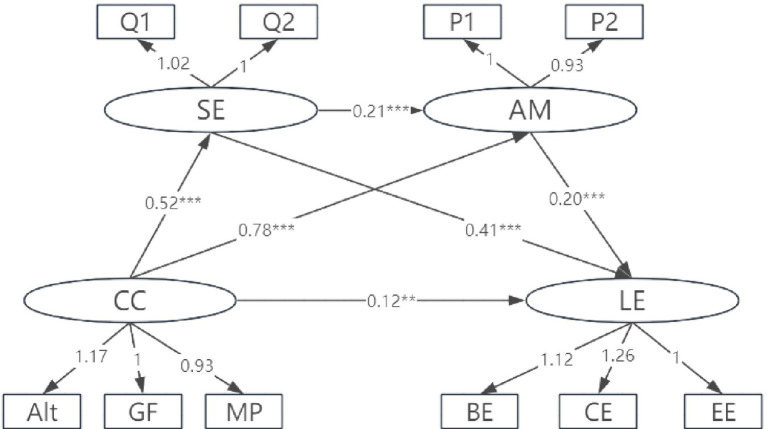
Chain mediation effects of achievement motivation and self-efficacy (female students). BE, behavioral engagement; CE, cognitive engagement; EE, emotional engagement; P1–P2, S1–S2, Q1–Q2 represent the packaged dimensions of achievement motivation, career calling, and self-efficacy, respectively.

In this study, there were significant gender differences in career calling (*M*_males_ > *M*_females_, *t´* = 2.39, *df* = 1,380, *p* = 0.017), achievement motivation (*M*_males_ > *M*_females_, *t´* = 6.45, *df* = 1,388, *p* < 0.001), and learning engagement (*M*_males_ > *M*_females_, *t´* = 3.43, *df* = 1,392, *p* < 0.001). Based on these differences, the study verified whether there was cross-cohort consistency in the chain mediation effects of self-efficacy and achievement motivation. First, the mediation models for male and female students were tested separately. The results showed that the fit indicators of the males’ model (*χ*^2^/*df* = 3.632, *χ*^2^ = 87.18, *df* = 24, CFI = 0.988, TLI = 0.978, GFI = 0.978, and RMSEA = 0.059) and the fit indicators of the females’ model (*χ*^2^/*df* = 3.825, *χ*^2^ = 91.81, *df* = 24, CFI = 0.989, TLI = 0.980, GFI = 0.985, RMSEA = 0.049) were acceptable for multi-group comparisons. Furthermore, unconstrained *M*_1_ and the equal measurement weight model *M*_2_ were set up. *M*_2_ constraints factor loadings to be equal across gender. A comparison of the fitting results of the two models revealed *χ*^2^/*df* = 4.80 (*χ*^2^ = 28.80 *df* = 6) and *p* < 0.001, indicating a significant difference between the two models (Table 3). The difference between ΔTLI and ΔCFI, the difference in the fitting indices of models *M*_1_ and *M*_2_, was less than 0.01 (Cheung and Rensvold, 2002), indicating that the respective equivalent models were valid. The infinite definite estimation models for men and women are shown in Figures 2, 3.

**Table 3:** Fitting indicators for gender differences in the mediation model.

Model	*M*_males_	*M*_females_	*M*_1_	*M*_2_	Recommended limits for model	Literature support	Interpretation of model fitness
*χ* ^2^ */df*	3.63	3.83	3.56	3.69	<3.00 (good)	Byrne (2010)	Within acceptable range, indicates good model fit.
					<5.00 (acceptable)		
TLI	0.98	0.98	0.98	0.98	≥0.95 (good)	Tucker and Lewis (1973)	Within good range, indicates good model fit.
					≥0.90 (acceptable)		
CFI	0.99	0.99	0.99	0.99	≥0.95 (good)	Bentler (1990)	Within good range, indicates good model fit.
					≥0.90 (acceptable)		
RMSEA	0.06	0.05	0.04	0.04	<0.05 (good)	Hu and Bentler (1999)	Within good range, indicates good model fit.
					<0.08 (acceptable)		
GFI	0.98	0.99	0.98	0.98	≥0.95 (good)	Jöreskog and Sörbom (1993)	Within good range, indicates good model fit.
					≥0.90 (acceptable)		
SRMR	0.03	0.03	0.03	0.04	<0.05 (good)	Hu and Bentler (1999)	Within good range, indicates good model fit.
					<0.08 (acceptable)		

AM, achievement motive; CC, career calling; SE, self-efficacy; LE, learning engagement.

Gender differences in the chain mediation effects of achievement motivation and self-efficacy were further examined using a non-parametric percentile bootstrap method. The results showed that the chain mediation model was consistent for both genders. The separate mediating effects of achievement motivation and self-efficacy, and the chain mediation of both, were significant. However, the effect values were inconsistent. These results suggest that the chain mediation model of achievement motivation and self-efficacy may have the same underlying structure in both genders, but with different implications.

The corresponding path coefficients of the structural equation modeling were further compared between male and female students. Significant gender differences were found in the path coefficients from achievement motivation to learning engagement (*γ*_male_ = 0.35, *γ*_female_ = 0.22, *c.r.* = −2.18, *p* < 0.001), while the other path coefficients are not significantly different (*c.r.* = −0.64 to 1.32, *p* > 0.05) (see Tables 4, 5).

**Table 4:** Estimates, SE, and gender differences in a mediating role.

Parameter	Male			Female		
	Estimates	SE	95% CI	Estimates	SE	95% CI
CC—SE—LE	0.23	0.04	[0.15, 0.32]	0.16	0.04	[0.09.0.24]
CC—AM—LE	0.24	0.05	[0.15, 0.35]	0.21	0.03	[0.15,0.29]
CC—SE—AM—LE	0.03	0.01	[0.02, 0.07]	0.02	0.01	[0.01,0.04]

**Table 5:** Hypothesis validation table.

Parameters (association)	Estimate value (*β*)	SE	Hypothesis support
CC–SE	0.37^***^	0.02	Yes
SE–LE	0.43^***^	0.04	Yes (Hypothesis 2)
CC–AM	0.49^***^	0.03	Yes
AM–LE	0.26^***^	0.03	Yes (Hypothesis 3)
SE–AM	0.27^***^	0.03	Yes (Hypothesis 4)
CC–LE (direct)	0.16^***^	0.05	Yes (partial effect)
Gender*AM–LE	−0.10^**^	0.04	Yes (moderation significant for males)

AM, achievement motive; CC, career calling; SE, self-efficacy; LE, learning engagement.

^**^p < 0.01, ^***^p < 0.001.

## 4 Discussion

In the 21st century, the aging of the global population is further aggravated, and international public health emergencies are occurring repeatedly. In this regard, the people of the world have put forward new requirements for public health safety, and governments have also increased their attention to medical education. As the primary body of medical education, the level of learning engagement of medical students is an important factor affecting the professionalism of medical students and an important indicator of the quality of national medical education (Huang et al., 2023). In response to the current problem of medical students’ learning engagement (Agarwal et al., 2019; Gómez et al., 2015; Xia et al., 2023; Xu et al., 2022), how to further improve the level of medical students’ learning engagement has become a problem that must be solved in the process of promoting the development of medical education. Meanwhile, the positive effects of career calling on individuals have been confirmed by numerous studies. Career calling has been found to be significantly positively correlated with learning engagement (Huang and Zhang, 2024), satisfaction (Laura et al., 2022), happiness (Duffy and Dik, 2013), job performance (Kim et al., 2018), self-efficacy (Yang et al., 2022), and achievement motivation (Yang et al., 2022). Therefore, the present study constructed a moderated chain mediation model with achievement motivation and academic self-efficacy as mediating variables and gender as a moderating variable to explore the effects and mechanisms of career calling on medical students’ learning engagement, which provided empirical evidence for subsequent effective interventions.

### 4.1 Relationship between career calling and learning engagement

The results of this study showed a significant positive correlation between career calling and learning engagement among medical students. That is, career calling significantly and positively predicted the learning engagement of medical students, thereby validating Hypothesis 1. This result is consistent with previous studies (Wang et al., 2023). The regression coefficient between career calling and learning engagement among medical students was 0.47, indicating a moderate effect size (Cohen, 1988). This suggests that a one-unit increase in career calling is expected to increase learning engagement by 0.47 units.

Individuals with a high sense of career calling had higher levels of learning engagement (Shang et al., 2022) or work engagement (Liu et al., 2019) and lower levels of academic burnout (He et al., 2023) or professional burnout (Li et al., 2023). This suggests that career calling is of great importance to medical students. It can positively predict the level of learning engagement and reduce academic burnout. Accordingly, improving medical students’ career calling and increasing their professional emotional experience can be an effective way to increase their learning engagement. In addition, the direct relationship between medical students’ career calling and learning engagement remained significant after the addition of the mediator variable, suggesting that the relationship may be aided by other factors.

### 4.2 Mediating role of self-efficacy

The results of this study showed that self-efficacy mediated the relationship between career calling and learning engagement, which verified Hypothesis 2. This result is in line with the previous results of Chen et al. (2016), who found that occupational self-efficacy had a mediating role between career calling and learning engagement when exploring the relationship between career calling and learning engagement of teacher educators based on the Social Cognitive Career Theory (SCCT). The same result was consistent with the findings of Shang et al. (2022). The regression coefficient between career calling and self-efficacy was 0.37, while that between self-efficacy and learning engagement was 0.43, both representing moderate effects (Cohen, 1988). This indicates that a 1-unit increase in career calling is expected to first increase self-efficacy by 0.37 units, then increase learning engagement by 0.16 units (0.37*0.43). The results of this study indicate that, when medical students have a stronger emotional experience of medical work, they tend to provide a more positive evaluation of their ability to complete their medical education. This positive evaluation will promote medical students’ performance in learning, that is, a higher level of learning engagement. The reason for this is that positive emotions have a positive effect on an individual’s cognitive appraisal (Nowlan et al., 2015). Positive appraisal not only improves an individual’s behavioral performance (Brühl et al., 2014) but also enhances their resilience to stress (Johnson et al., 2010) and boosts their persistence in completing tasks. The results of this study support the SCCT prediction and further enrich related research by demonstrating that career calling and self-efficacy are important factors influencing learning engagement in the medical student population.

### 4.3 The mediating role of achievement motivation

The results of this study showed that achievement motivation mediated the relationship between career calling and learning engagement, thereby validating Hypothesis 3. The results of this study revealed that achievement motivation mediated the relationship between career calling and learning engagement, which verified Hypothesis 3. There is a lack of existing studies that directly investigate the relationship between achievement motivation, career calling, and learning engagement, and the results of the present study further enrich the relevant research. The regression coefficient between achievement motivation and career calling was 0.49, indicating a moderate-to-strong effect. The regression coefficient between achievement motivation and learning engagement was 0.26, representing a small to moderate effect (Cohen, 1988). An increase of 1 unit in career calling is expected to first increase achievement motivation by 0.49 units and then increase learning engagement by 0.13 units (0.49*0.26). The findings suggest that, when medical students have a strong sense of career calling, they take learning as an effective path to achieve their career goals, which enables them to experience the value and significance of learning in the process of learning, and this sense of value further increases the achievement motivation of medical students. The higher the achievement motivation, the more individuals desire to achieve academic success, and naturally, they are more willing to increase their learning engagement (Atkinson, 1957). This performance is consistent with the expectancy-value theory, which states that, when individuals can perceive the value of a task, this value enhances their motivation to do what they are supposed to do, that is, it enhances their achievement motivation (Eccles and Wigfield, 2002). As achievement motivation increases, individuals are willing to put in more effort when faced with challenges. The results of the present study enrich the relevant research on expectancy-value theory by demonstrating that achievement motivation is an important factor influencing the relationship between career calling and learning engagement in the medical student population. This suggests that enhancing the achievement motivation of medical students can be used as one of the effective ways to improve their learning engagement.

### 4.4 Chain mediation of perceived achievement motivation and academic self-efficacy

The findings showed that achievement motivation and learning self-efficacy acted as chain mediators between career calling and learning engagement, verifying Hypothesis 4. This provides a new perspective for understanding the relationship between career calling and learning engagement. The regression coefficient between self-efficacy and achievement motivation was 0.27, indicating a small to medium effect size (Cohen, 1988). An increase of 1 unit in self-efficacy is expected to increase achievement motivation by 0.27 units. There was a significant positive correlation between achievement motivation and self-efficacy, consistent with previous studies (Cheng et al., 2022; Li et al., 2021; Luo et al., 2023). According to expectancy-value theory (Wigfield and Eccles, 2000), achievement motivation is influenced by expectations, which are individuals’ subjective judgments about their likelihood of successfully completing a task. Therefore, individuals with high self-efficacy have a positive judgment about their likelihood of successfully completing a task, and their achievement motivation is naturally higher. In summary, career calling leads individuals to experience positive emotions when engaging in career behaviors, and such positive emotions first promote individuals’ self-efficacy (Hu et al., 2024). Achievement motivation is influenced by self-efficacy, which is followed by an upward trend (Wigfield and Eccles, 2000). Finally, as the achievement motivation of medical students increases, their need for academic success subsequently increases, which in turn prompts medical students to study harder and increases their level of learning engagement (Atkinson, 1957). The results of the present study extend the research related to learning engagement in that career calling, as a distal factor, can not only directly influence learning engagement but also indirectly, through the combined effect of two proximal factors, influence self-efficacy and achievement motivation.

### 4.5 Moderating role of gender

The study found that boys and girls remained consistent on the mediating paths of achievement motivation and academic self-efficacy through the multiple cohort test, but there were differences in effect sizes. Further comparison of the differences in path coefficients revealed significant gender differences in the path coefficients from achievement motivation to learning engagement, with the addition of the two mediating variables. The path coefficients for male students were significantly larger than those for female students. The path coefficient from achievement motivation to learning engagement for men was 0.3, indicating a moderate effect size. For women, the path coefficient was 0.2, representing a small to moderate effect (Cohen, 1988). A significant difference was observed between the two groups. The reason for this may stem from the fact that different genders are assigned different social role expectations (Jennifer and Paul, 1999). For men, society expects them to carry the burden of being the primary breadwinner for the family and to be professionally successful (Lesser et al., 1963). This not only affects men’s perceptions of success but also their specific behaviors. Specifically, male students will use academic success as their criterion for success, and to fulfill this criterion, they will further increase their learning engagement. In contrast, for women, society expects them to take care of their families more, emphasizing their importance in the family and marriage (Wei et al., 2024). In response, female students may fear that academic success is perceived by others as an unsuitable success goal for women, whose success criteria may be more complex (Horner, 1968). Consequently, male students were more determined to gain achievement experiences through increased learning engagement than female students. This is in line with the results of the higher levels of learning engagement and achievement motivation of male students in the current study. This suggests that the impact of gender should be considered when training medical students. Female students first need to be helped to develop a proper sense of success, after which their learning engagement can be further enhanced.

### 4.6 Implications and perspectives

This study revealed that self-efficacy and achievement motivation exert a chain-mediated effect on the relationship between career calling and learning engagement. This study has both theoretical significance and practical value.

Theoretically, it first helps elucidate the mechanism through which career calling influences learning engagement, clarifying the indirect pathway via self-efficacy and achievement motivation. Second, it enriches the content of the Social Cognitive Career Theory. This study integrates the role of career calling in individual career development into a systematic framework, demonstrating that career calling can serve as an antecedent variable influencing efficacy and outcome expectations, ultimately affecting individual career performance.

Practically, exploring the influence mechanism of learning engagement from the perspective of career calling provides an effective pathway for further enhancing medical students’ learning engagement and improving the quality of medical education. First, cultivating medical students’ career calling will help increase their level of learning engagement. A strong career calling helps medical students clarify their learning objectives, discover the value and meaning of learning, and internalize their learning motivation. Second, the mediating role of self-efficacy and achievement motivation suggests that, when cultivating medical students’ career calling, we should also guide this strong calling toward individual efficacy and outcome expectations. This fosters the development of self-efficacy and achievement motivation in students. As medical students increasingly desire academic success and continuously improve their self-assessment of academic task completion abilities, they will progressively enhance their learning engagement levels, achieve academic success, and enter a positive learning cycle. This underscores the need to develop counseling and training programs in medical education that address self-efficacy, career calling, and achievement motivation, thereby helping students to further elevate their learning engagement. Finally, in practical educational settings, we must recognize the impact of gender on medical students’ learning engagement. Efforts should be made to help female students establish a healthy perception of success, strengthen the connection between achievement motivation and learning engagement, and ensure that all students can focus their energy on their studies.

Inevitably, this study has room for improvement. First, the cross-sectional design employed in this study fails to establish causal relationships among the variables under study. Future studies should incorporate longitudinal designs to further examine the directionality and developmental trajectories of these variables. Second, the sample consisted solely of medical students from two Chinese universities, potentially limiting its representativeness and affecting the external validity of our findings. Future studies could broaden the sampling scope by employing multistage sampling to cover medical students across multiple countries, thereby enhancing the external validity of the findings. Finally, this study relied solely on self-reporting as an observational metric, making it susceptible to social desirability effects. Subsequent studies should collect data from multiple perspectives, such as individual, parental, and teacher evaluations, or incorporate additional objective metric data to mitigate this limitation.

## 5 Conclusion

1. Achievement motivation and self-efficacy mediate the relationship between career calling and learning engagement.
2. Achievement motivation and self-efficacy act as chain mediators between career calling and learning engagement.
3. In the chain mediation model, the effect of achievement motivation on learning engagement was moderated by gender.

## Data Availability

The raw data supporting the conclusions of this article will be made available by the authors, without undue reservation.
